# Ultra-dense optical data transmission over standard fibre with a single chip source

**DOI:** 10.1038/s41467-020-16265-x

**Published:** 2020-05-22

**Authors:** Bill Corcoran, Mengxi Tan, Xingyuan Xu, Andreas Boes, Jiayang Wu, Thach G. Nguyen, Sai T. Chu, Brent E. Little, Roberto Morandotti, Arnan Mitchell, David J. Moss

**Affiliations:** 1 Photonic Communications Lab, Department of Electrical and Computer System Engineering, Monash University, Clayton, VIC 3168 Australia; 2 Centre for Micro-Photonics, Swinburne University of Technology, Hawthorn, VIC 3122 Australia; 3 Integrated Photonics and Applications Centre (InPAC), School of Engineering, RMIT University, Melbourne, VIC 3001 Australia; 4 Department of Physics and Material Science, City University of Hong Kong, Tat Chee Avenue Hong Kong, China; 5 Xi’an Institute of Optics and Precision Mechanics Precision Mechanics of CAS, Xi’an, China; 6 INRS-Énergie, Matériaux et Télécommunications, 1650 Boulevard Lionel-Boulet, Varennes Québec, J3X 1S2 Canada; 7 Institute of Fundamental and Frontier Sciences, University of Electronic Science and Technology of China, Chengdu, 610054 China

**Keywords:** Electrical and electronic engineering, Fibre optics and optical communications, Frequency combs, Solitons

## Abstract

Micro-combs - optical frequency combs generated by integrated micro-cavity resonators – offer the full potential of their bulk counterparts, but in an integrated footprint. They have enabled breakthroughs in many fields including spectroscopy, microwave photonics, frequency synthesis, optical ranging, quantum sources, metrology and ultrahigh capacity data transmission. Here, by using a powerful class of micro-comb called soliton crystals, we achieve ultra-high data transmission over 75 km of standard optical fibre using a single integrated chip source. We demonstrate a line rate of 44.2 Terabits s^−1^ using the telecommunications C-band at 1550 nm with a spectral efficiency of 10.4 bits s^−1^ Hz^−1^. Soliton crystals exhibit robust and stable generation and operation as well as a high intrinsic efficiency that, together with an extremely low soliton micro-comb spacing of 48.9 GHz enable the use of a very high coherent data modulation format (64 QAM - quadrature amplitude modulated). This work demonstrates the capability of optical micro-combs to perform in demanding and practical optical communications networks.

## Abstract (web summary)

Microcombs provide many opportunities for integration in optical communications systems. Here, the authors implement a soliton crystal microcomb as a tool to demonstrate more than 44 Tb/s communications with high spectral efficiency.

## Introduction

The global optical fibre network currently carries hundreds of terabits per second every instant, with capacity growing at ~25% annually^1^. To dramatically increase bandwidth capacity, ultrahigh capacity transmission links employ massively parallel wavelength division multiplexing (WDM) with coherent modulation formats^2,3^, and in recent lab-based research, by using spatial division multiplexing (SDM) over multicore or multi-mode fibre^4^. At the same time, there is a strong trend towards a greater number of shorter high-capacity links. Whereas core long-haul (spanning 1000’s km) communications dominated global networks 10 years ago, now the emphasis has squarely shifted to metro-area networks (linking across 10’s–100’s km) and even data centres (< 10 km). All of this is driving the need for increasingly compact, low-cost and energy-efficient solutions, with photonic integrated circuits emerging as the most viable approach. The optical source is central to every link, and as such, perhaps has the greatest need for integration. The ability to supply all wavelengths with a single, compact integrated chip, replacing many parallel lasers, will offer the greatest benefits^5,6^.

Micro-combs, optical frequency combs based on micro-cavity resonators, have shown significant promise in fulfilling this role^7–10^. They offer the full potential of their bulk counterparts^11,12^, but in an integrated footprint. The discovery of temporal soliton states (DKS—dissipative Kerr solitons)^10,13–17^ as a means of mode-locking micro-combs has enabled breakthroughs in many fields including spectroscopy^18,19^, microwave photonics^20^, frequency synthesis^21^, optical ranging^22,23^, quantum sources^24,25^, metrology^26,27^ and more. One of their most-promising applications has been optical fibre communications, where they have enabled massively parallel ultrahigh capacity multiplexed data transmission^28–30^.

The success of micro-combs has been enabled by the ability to phase-lock, or mode-lock, their comb lines. This, in turn, has resulted from exploring novel oscillation states such as temporal soliton states, including feedback-stabilised Kerr combs^29^, dark solitons^30^ and DKS^28^. DKS states, in particular, have enabled transmission rates of 30 Tb/s for a single device and 55 Tb/s by combining two devices, using the full C and L telecommunication bands^28^. In particular, for practical systems, achieving a high spectral efficiency is critically important—it is a key parameter as it determines the fundamental limit of data-carrying capacity for a given optical communications bandwidth^2,3^.

Recently^17,11^, a powerful class of micro-comb termed soliton crystals was reported, and devices realised in a CMOS (complementary metal-oxide semiconductor) compatible platform^2,3,8,9,31^ have proven highly successful at forming the basis for microwave and RF photonic devices^32,33^. Soliton crystals were so-named because of their crystal-like profile in the angular domain of tightly packed self-localised pulses within micro-ring resonators (MRRs)^17^. They are naturally formed in micro-cavities with appropriate mode-crossings without the need for complex dynamic pumping and stabilisation schemes that are required to generate self-localised DKS waves (described by the Lugiato-Lefever equation^34^). The key to their stability lies in their intracavity power that is very close to that of spatiotemporal chaotic states^17,35^. Hence, when emerging from chaotic states there is very little change in intracavity power and thus no thermal detuning or instability, resulting from the ‘soliton step’ that makes resonant pumping more challenging^36^. It is this combination of intrinsic stability (without the need for external aid), ease of generation and overall efficiency that makes them highly suited for demanding applications such as ultrahigh-capacity transmission beyond a terabit/s.

Here, we report ultrahigh bandwidth optical data transmission across standard fibre with a single integrated chip source. We employ soliton crystals realised in a CMOS-compatible platform^31–33^ to achieve a data line-rate of 44.2 Tb/s from a single source, along with a high spectral efficiency of 10.4 bits/s/Hz. We accomplish these results through the use of a high modulation format of 64 QAM (quadrature amplitude modulation), a low comb-free spectral range (FSR) spacing of 48.9 GHz, and by using only the telecommunications C-band. We demonstrate transmission over 75 km of fibre in the laboratory as well as in a field trial over an installed network in the greater metropolitan area of Melbourne, Australia. Our results stem from the soliton crystal’s extremely robust and stable operation/generation as well as its much higher intrinsic efficiency, all of which are enabled by an integrated CMOS-compatible platform.

## Results

### Experiment

**Fig. 1:**
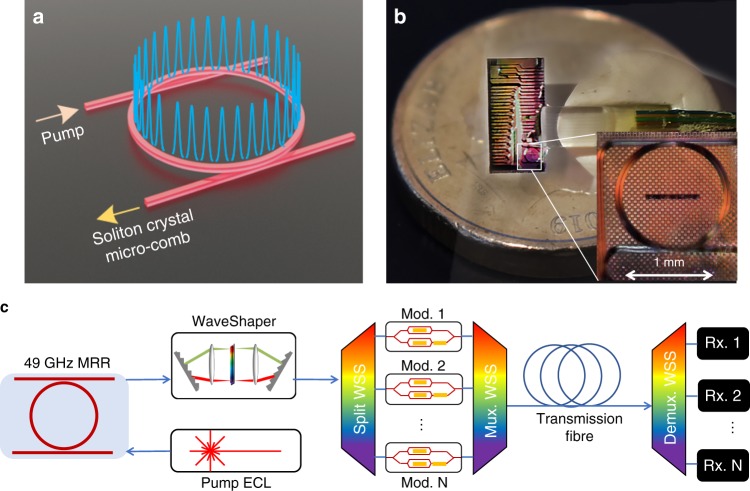
Conceptual diagram of a soliton crystal micro-comb communications experiment. **a**. Illustration of the soliton crystal state used in this paper. We infer from the generated spectrum that the state was a single temporal defect crystal across the ring. The state had a characteristic ‘scalloped’ micro-comb spectrum, corresponding to the single temporal defect crystal state. **b** Photograph of the fibre-optic packaged micro-ring resonator chip used for soliton crystal generation. The full chip is 5 mm × 9 mm, of which we use devices and access waveguides on ~  ¼ of the area. The AUD $2 coin (20.5 mm diameter) shown for scale is similar in size to a USD nickel or a 10 Euro cent coin. Inset is a microscope image of the ring resonator element, with a scale bar. Visible distortions are due to an overlayer of glue from the fibre array. **c** Experimental setup. A CW laser, amplified to 1.8 W, pumped a 48.9 GHz FSR micro-ring resonator, producing a micro-comb from a soliton crystal oscillation state. The comb was flattened and optically demultiplexed to allow for modulation, and the resulting data optically multiplexed before the subsequent transmission through fibres with EDFA amplification. At the receiver, each channel was optically demultiplexed before reception. *ECL* edge-coupled laser, *WSS* wavelength-selective switch, *Rx* receiver.

**Fig. 2:**
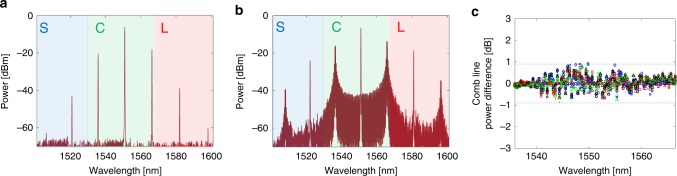
Soliton crystal generation. To generate a soliton crystal, a laser is slowly tuned from the red side of a resonance to a pre-set wavelength. **a** A primary comb (Turing pattern) is initially generated as the laser is tuned into resonance with the ring. **b** Spectrum of the soliton crystal oscillation state used for experiments. The state had a characteristic ‘scalloped’ micro-comb spectrum, corresponding to the single temporal defect crystal state illustrated over the ring. At the pre-set wavelength, a soliton crystal forms, with spectral features based around the primary comb lines. The state that we use provides comb lines over most of the optical communications C-band. **c** Soliton crystal comb line power difference for 10 independent crystal generation instances (different symbols indicate distinct generation instances). Comb line powers remain within ± 0.9 dB of the initial spectrum, indicating reliable generation of the desired soliton crystal state.

A schematic illustrating the soliton crystal optical structure is shown in Fig. 1a, with the physical chip shown in Fig. 1b and the experimental setup for ultrahigh bandwidth optical transmission in Fig. 1c (also see Methods and Supplementary Note 1). The micro-resonator had an FSR spacing of 48.9 GHz and generated a soliton crystal with this spacing (~0.4 nm) over a bandwidth of >80 nm when pumped with 1.8 W of continuous-wave (CW) power (in-fibre, incident) at 1550 nm. The soliton crystal micro-comb was generated by automatically tuning the pump laser to a pre-set value. The primary comb and generated soliton crystal states are shown in Figs. 2a and b. Figure 2c demonstrates the stability of the soliton crystal comb generation by showing a variation in individual tone powers of < ± 0.9 dB over 10 different generation instances through wavelength sweeping (from 1550.300 to 1550.527 nm). This demonstrates the repeatability of turn-key micro-comb generation from this integrated CMOS-compatible device.

From the generated micro-comb, 80 lines were selected over the telecommunications C-band (32 nm wide, 3.95 THz window from 1536 to 1567 nm), which were then flattened with a spectral shaper (WaveShaper 4000 S—see Methods). Next, the number of wavelengths were effectively doubled to 160 (equivalent to a comb spacing of 24.5 GHz) to optimise the spectral efficiency (spectrally useful content) by using a single-sideband modulation scheme to generate odd/even de-correlated test channels (see Methods). We then combined a test band of six channels, with the remaining bands providing loading channels having the same odd-and-even channel structure. We used a high order format of 64 QAM to modulate the entire comb at a symbol rate of 23 Gigabaud, resulting in the utilisation of 94% of the available spectrum.

**Fig. 3:**
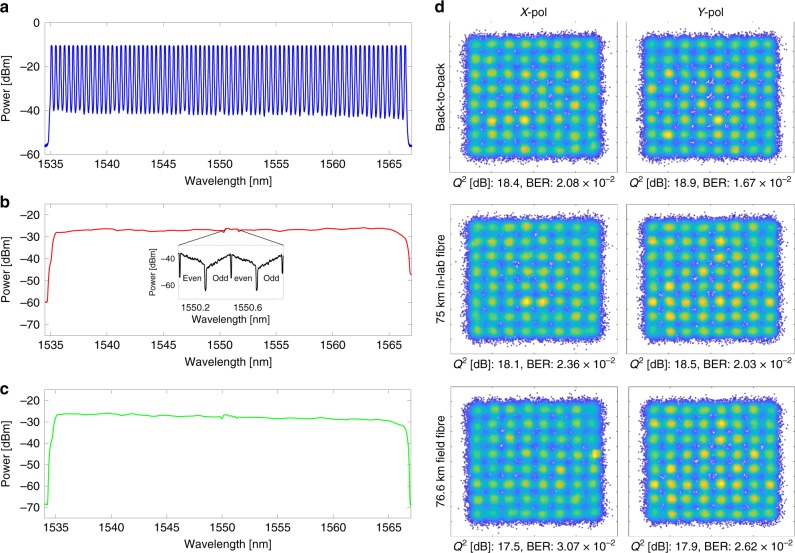
Soliton crystal super-channel spectra, and indicative signal constellations. **a**–**c** Spectra of the soliton crystal frequency comb after flattening **a**, modulation and transmission through either 75 km spooled in-lab fibre **b** or through the field-trial link **c**. The spectrum **a** is measured with 12.5 GHz resolution to resolve the individual comb lines, whereas **b** and **c** are plotted at 50 GHz resolution to illustrate average channel powers. Flattening equalised the comb line power to within 1 dB. After modulation and amplification, the channels were shaped by the EDFA gain spectrum. The inset in **b** depicts the test channel spectra captured with a 150 MHz resolution optical spectrum analyser (Finisar WaveAnalyzer), highlighting the odd and even sub-bands modulated onto each comb line in the test band. **d** Constellation diagrams for a comb line at 193.4 THz (1550.1 nm) for both X- and Y-polarisation channels. ‘Back-to back’ denotes the transmitter directly connected to the receiver, ’75 km in-lab fibre’ indicates reception after transmission through 75 km of spooled fibre inside the lab (as per **b**), whereas ’76.6 km field fibre’ denotes reception after transmission through the field-trial link (as per **c**). BER and *Q*^2^ related to the constellations are noted on each.

We conducted two transmission experiments, sending data over 75 km of single-mode fibre in the laboratory as well as in a field trial across an installed metropolitan area single-mode fibre network (see Supplementary Note 1) connecting the Melbourne City campus of RMIT and the Clayton campus of Monash University, spanning the greater metropolitan area of Melbourne. Spectra of the comb at key points are given in Fig. 3a–c. At the receiver, the signal was recovered using a common offline digital signal processing (DSP) flow (see Methods). Figure 3d shows constellation diagrams for the signal at 194.34 THz. In the back-to-back configuration (i.e., with the transmitter directly connected to the receiver) we measured a signal quality (*Q*^2^, from error-vector magnitude) approaching 18.5 dB, dropping to near 17.5 dB when transmitting the fully modulated comb through the test links.

### Transmission results

**Fig. 4:**
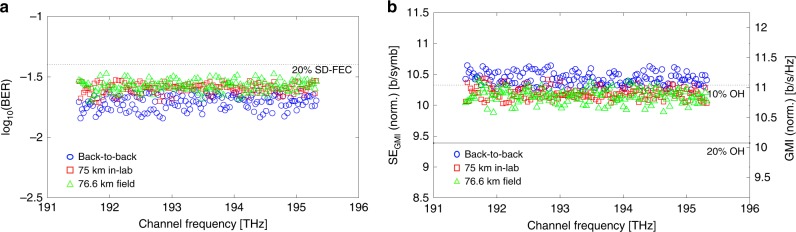
Bit-error ratio, spectral efficiency and GMI for transmission experiment. **a** BER for each comb line. Blue circle points indicate the performance of the channels in a B2B configuration, red square dots are the performance after transmission through 75 km of in-lab spooled fibre, whereas the green triangles are after transmission through the 76.6 km installed metropolitan area fibre link. An indicative FEC threshold is given at 4 × 10^−2^, corresponding to a pre-FEC error rate for a 20% soft-decision FEC based on spatially-coupled LDPC codes^2^ (dashed line). After transmission, all channels were considered to be error-free. **b** GMI and spectral efficiency measured for each comb line. GMI was calculated after normalisation to scale the measured constellations in order to account for the received signal-to-noise ratio (SNR). Lines are for 20% and 10% overheads. Spectral efficiency was derived from GMI, and the ratio of symbol rate to comb spacing. GMI indicates a higher overall capacity than BER with the indicated SD-FEC threshold, as GMI assumes the adoption of an ideal code for the system. For B2B, GMI (SE) varied between 11.3 b/symb. (10.6 b/s/Hz) and 10.9 b/symb. (10.3 b/s/Hz). After in-lab fibre transmission, the achievable per-channel GMI (SE) varied between 11.0 b/symb. (10.4 b/s/Hz) and 10.7 b/symb. (10.1 b/s/Hz), with the same range observed for the installed field-trial fibres. We estimate the overall capacity from the sum of the GMIs, multiplied by the symbol rate.

Figure 4a shows the transmission performance using the bit-error ratio (BER) for each channel as a metric. Three scenarios were investigated: (i) a direct connection between the transmitter stage to the receiver (back-to-back, B2B) and after transmission through (ii) in-lab fibre and (iii) over the field trial network. Transmission globally degraded the performance of all channels, as expected. As a performance benchmark, Fig. 4a indicates a 20% soft-decision forward error correction (SD-FEC) threshold given at a BER of 4 × 10^−2^ from a demonstrated code^37^. All results were below the given FEC limit, but since using SD-FEC thresholds based on BER is less accurate for higher-order modulation formats and for high BERs^38^, we additionally used generalised mutual information (GMI) to calculate the system performance. Figure 4b plots the GMI for each channel and its associated SE, with lines given to indicate projected overheads. We achieved a raw bitrate (line-rate) of 44.2 Tb/s, which translates to an achievable coded rate of 40.1 Tb/s (in B2B), dropping to 39.2 Tb/s and 39.0 Tb/s for the lab and field-trial transmission experiments, respectively. These yielded spectral efficiencies of 10.4, 10.2, and 10.1 b/s/Hz (see Methods). These data rates represent an increase of ~50% (see Methods) over the highest reported result from a single integrated device^28^, whereas the spectral efficiency is 3.7 times higher. This is notable considering that the experiments were performed with the added penalty of full comb flattening (equalisation, even though this is in fact not necessary^39^), and without closed-loop feedback or stabilisation.

## Discussion

Table 1 summarises key results from the literature comparing the various system performance metrics for demonstrations based on a single integrated source and over standard fibre (or calculated on a per-mode basis for multicore fibre). Previous to this work, the best result (per core)^28^ was based on single micro-comb that was able to support 30.1 Tb/s over the C and L bands, when using a standard tuneable laser coherent receiver. This is the benchmark result that we compare our results to since it is not only the best published result using a single micro-comb, but it closely resembles our experiment (single micro-comb at the transmitter, single tuneable laser at the receiver as a local oscillator). Note that our system uses less than half the spectrum of ref. ^28^, whereas substantially exceeding its data rate, owing to our much higher spectral efficiency (3.7× higher). High modulation formats have also been achieved with dark solitons^30^, yet at a lower overall data rate, primarily owing to the high comb line spacing that significantly limits the spectral efficiency. The work of ref. ^4^ used a comb generator based on a benchtop pulsed seed fibre laser source combined with waveguide spectral broadening. To provide a fully integrated system, this source would need to be on-chip. The focus in that experiment was using novel, proprietary multicore fibre to achieve a 30-fold increase in bandwidth over the fibre in this spatially multiplexed system, to reach 0.66 Petabits/s. On a per-mode basis^4^ this yields 25.6 Tb/s/mode, a lower per-mode capacity than this work and that of ref. ^28^. We note that both our approach and that of ref. ^28^ are able to take advantage of SDM techniques to scale the overall bandwidth by using multicore fibre. We provide further comparisons in the Supplementary Note 3.

**Table 1:** Key systems performance metrics.

Line rate	Net rate	Format	Spectral efficiency	Transmission	Source
30.1 Tb/s	28.0 Tb/s	16 QAM	2.8 b/s/Hz	75 km SMF in-lab	Ref. ^28^
4.8 Tb/s*	4.4 Tb/s	64 QAM	1.1 b/s/Hz*	80 km SMF in-lab	Ref. ^30^
25.6 Tb/s^1^	22.0 Tb/s^1^	16 QAM	3.2 b/s/Hz^1^	9.6 km, 30-core fibre^1^	Ref. ^41^
44.2 Tb/s	40.1 Tb/s	64 QAM	10.4 b/s/Hz	B2B (0 km)	This work
44.2 Tb/s	39.2 Tb/s	64 QAM	10.2 b/s/Hz	75 km SMF in-lab	This work
44.2 Tb/s	39.0 Tb/s	64 QAM	10.1 b/s/Hz	76.6 km SMF installed	This work

^1^Results from ref. ^4^ were based on a non-integrated benchtop laser source and are shown for reference. For this space division multiplexing (SDM) demonstration we quote numbers that are per spatial mode.

*indicates that this figure was not directly provided in the reference, and so is inferred from data provided.

Our high transmission capacity and spectral efficiency are partly a result of the high conversion efficiency between the injected CW wave and the soliton crystal state^17,33^, which is reflected in the near absence of a soliton step (the jump in intracavity power that often occurs when generating coherent soliton states).

Although our experiments were restricted to the C-band, the soliton crystal comb (Fig. 2b) had a bandwidth exceeding 80 nm. The comb lines in the S and L bands (1500–1535 nm and 1565–1605  nm) could in principle be increased in power to enable transmission across all three bands, by varying the pump wavelength and power, tailoring dispersion, and/or by other methods. Assuming similar comb quality, this would result in a threefold increase in total data rate to 120 Tb/s from a single integrated device.

Micro-combs with lower FSRs would support even higher spectral efficiencies since the signal quality improves at lower symbol rates. However, this may come at the expense of a narrower overall comb bandwidth. In our demonstration, single-sideband modulation enabled the multiplexing of two channels onto a single light source, effectively halving the comb spacing while improving back-to-back performance as limited by transceiver noise. This approach is made feasible by the stable nature of the soliton crystal combs. On the other hand, electro-optic modulation can also be used to sub-divide the repetition rate of micro-combs, which would enable broad comb-bandwidths. Although this would require locking the comb spacing to an external RF source, sub-MHz stabilisation of comb spacings has been reported^40,41^. Further, boosting the comb generation efficiency through laser cavity-soliton micro-combs^42^ may provide a powerful path to improve signal quality and system capacity even further. Finally, for newly deployed links, our approach can readily be combined with space division multiplexing using multicore fibre^4,43^, which would result in data rates of many petabit/s from a single source.

In conclusion, we report high-performance ultrahigh bandwidth optical transmission from a single integrated chip source by using soliton crystal micro-combs. This achievement is a result of the low comb spacing combined with the stable, efficient, and broad bandwidth of the soliton crystal combs, all enabled by their CMOS-compatible integration platform. Soliton crystal micro-combs are intrinsically coherent, low noise, and can be initialised and maintained using standard open-loop control with off-the-shelf equipment. This work demonstrates their ability to support ultrahigh bandwidth data transmission in practical and demanding environments.

## Methods

### CMOS-compatible micro-comb source

The MRR for comb generation was fabricated using CMOS-compatible processes^10,30,31^ with doped silica glass waveguides, which features low linear loss (~0.06 dB cm^−1^), a moderate nonlinear parameter (~233 W^−1^ km^−1^), and negligible nonlinear loss that leads to an ultrahigh nonlinear figure of merit. The MRR has a cross-section of 3 × 2 μm and a radius of ~592 μm, yielding an FSR of 48.9 GHz and a Q factor > 1.5 million. The dispersion of the TM mode was designed to be anomalous in the C band with a jump at ~ 1552 nm brought about by the mode crossing. The bus waveguides of the MRR were directed to on-chip mode converters, then coupled to a single-mode fibre array, with a fibre-chip coupling loss of ~0.5 dB per facet.

Although statistical studies of fabrication yield are outside the scope of this work, we note that our platform is fully CMOS-compatible, using stepper mask aligners on full wafers^44^. Further, our low index contrast (core index = 1.7), results in larger waveguide dimensions, which in turn makes them less sensitive to fabrication error. Our typical yields for FSR and Q factor are extremely high—well above 90%, and mode-crossings do not pose a particular challenge. We have fabricated many soliton crystal devices^35^ with high reproducibility. The discovery that mode-crossings provide a powerful approach towards achieving robust or even deterministic generation of micro-combs shows that further engineering of these structures remains an important and highly beneficial challenge that will yield new functionality.

### Soliton crystal micro-comb generation

The micro-comb used in the study was generated from the doped silica double-bus micro-ring resonator described above, packaged with a fibre array connection to all four device ports. We pumped the ring with a CW external cavity laser (Yenista Tunics—100S-HP) at an output power of 15 mW, which was then amplified to 1.8 W in a polarisation maintaining erbium doped fibre amplifier (EDFA) (Pritel PMFA-37). Only the TM mode of the resonator oscillated in a soliton crystal state, hence the pump polarisation was tuned to match this mode. As indicated in Fig. 1a, we inserted the pump light into the ‘through’ port and collected light from the corresponding ‘drop’ port. The MRR chip was mounted on a Peltier cooler, monitored by a standard NTC temperature sensor. The temperature was maintained with a thermo-electric cooler (TCM-M207) at 25˚C, within 0.1˚C of accuracy. The laser was set to standard running mode, with no extra steps made to stabilise the output frequency. Soliton crystal generation was achieved by automated wavelength tuning, in turn reducing the system complexity compared with other micro-comb generation schemes (see ref. ^12^ and references within). We measured the internal conversion efficiency of our soliton crystals to be 42% for the whole spectrum, and 38% when selecting the 80 lines over the C-band, highlighting that over 90% of our available comb power is compatible with standard C-band equipment (see Supplementary Note 2).

The generated soliton crystal micro-comb was then flattened in two stages by two independent programmable optical filters (Finisar WaveShaper 4000 S). The WaveShapers had an insertion loss of 5 dB each, in addition to any variable attenuation. The first had a static filter shape set to equalise each comb line to within ~1 dB of each other, to coarsely match the generic shape of the soliton crystal state we chose to use. The second programmable filter was set each time that a new soliton crystal state was initiated, to equalise the comb line powers to within < 1 dB of each other, although we note that it was often unnecessary to change the filter profile when generating a new soliton crystal. Spectral shaping in a WDM transceiver using a comb source involved minimal extra complexity as only the addition of attenuators after the WDM demultiplexer was required to route each comb line to a separate modulator. The comb was then amplified by a further polarisation maintaining EDFA (Pritel PMFA-20-IO), before being divided for modulation. Prior to modulation, the optical signal-to-noise ratio (OSNR) of the individual comb lines was > 28 dB (see Supplementary Note 2).

The nonuniform spectrum of soliton crystal combs has been considered as a drawback, and so for this reason, as well as to facilitate easier comparison with prior work using micro-combs, we ensured that the optical frequency comb was flattened such that all lines were of equal power.

Comb flattening in fact is not necessary either in our experiments or other micro-comb demonstrations (e.g., ^28,30^.), as all comb lines are typically wavelength demultiplexed into separate waveguides and sent to separate modulators. It is then straightforward to adjust the comb line power by variable attenuators, amplifiers, or even by varying the RF drive amplitude to the modulators. In fact, we expect better performance without comb flattening, as the higher power comb lines would need less attenuation and/or amplification before modulation, resulting in a higher OSNR, whereas the lower power comb lines would have essentially the same performance as reported here. Furthermore, using the raw spectrum would avoid the loss of the extra Waveshaper. Therefore, avoiding flattening (working with the raw spectrum) would in fact yield even higher system performance.

### Systems experiment

The detailed experimental setup is shown in Supplementary Fig. 1. The transmitter used three separate complex Mach-Zehnder modulators to provide both odd and even test bands, as well as a loading band. The comb lines for each of these bands were split using another programmable filter (Finisar WaveShaper 4000 S) and were then amplified before modulation. Three tones separated by 98 GHz around the selected test frequency were directed to two separate modulators (Sumitomo Osaka Electric Company New Business T.SBXH1.5-20). The two modulators were driven at a symbol rate of 23 Gbaud, providing a per sub-band line rate (i.e., excluding overheads) of 23 Giga-symbols/s × 6 bits/symbol × 2 polarisations = 276 Gb/s. The sub-bands were shifted by 12 GHz from the optical carrier, with one modulator providing a sideband down-shifted from the optical carrier, and the other an up-shifted sideband. This enabled higher fidelity modulation than simple 46 Gbd single-carrier modulation, given the transceiver noise limitations we had in our system. The odd and even bands were de-correlated by adding a delay with an extra length of optical fibre in the ‘even’ path. A third modulator (Covega Mach-40 806) was used to modulate the loading bands, which consisted of a two Nyquist sub-carrier modulation scheme to mimic the structure of the odd and even test bands. The two bands were driven by pairs of the positive and negative differential outputs of the AWG (Keysight M8195A, 65 GSa/s, 25 GHz bandwidth), whereas the loading channels were driven by a separate independent output pair. The modulating waveforms were set to provide 64 QAM signals, pulse shaped by a 2.5% roll-off RRC filter, running at 23 Gigabaud. On a 48.9 GHz grid, this provided a 94% spectral occupancy. The modulator optical outputs were each passed through a polarisation maintaining 3 dB power splitter, one output being delayed by a few metres of optical fibre and then rotated by 90^o^ using a polarisation beam splitter/combiner. This provided emulation of polarisation multiplexing by delay de-correlation. The odd, even and loading bands were all de-correlated from each other by means of different fibre delays of a few metres. The odd and even channels were passively combined with a 3-dB power splitter, to maintain the pulse shape of the central channels. The combined test and loading bands were multiplexed by a further programmable filter (Finisar WaveShaper 4000 S). The roll-off of the filters from this device did affect the outer channels of the test band and the neighbouring channels in the loading channels. After multiplexing the fully modulated comb was amplified to a set launch power. The Tx DSP is described in Supplementary Note 1.

The physical fibre-optic network geography is shown in Supplementary Fig. 2 and the schematic layout in Supplementary Fig. 1 (see Supplementary Note 1). The transmission link was comprised of two fibre cables connecting labs at RMIT University (Swanston St., Melbourne CBD) and Monash University (Wellington Rd, Clayton). These cables were routed from the labs access panels, to an interconnection point with the AARNet’s fibre network. The fibre links are a mix of OS1 and OS2 standard cables and include both subterranean and aerial paths. There is no active equipment on these lines, providing a direct dark fibre connection between the two labs. The total loss for these cables was measured to be 13.5 dB for the RMIT-Monash link and 14.8 dB for the Monash-RMIT paths. The cable lengths as measured by OTDR were both 38.3 km (totalling 76.6 km in loop-back configuration). At Monash, an EDFA was remotely monitored and controlled using a 1310 nm fibre-ethernet connection running alongside the C-band test channels. The comb was amplified to 19 dBm before launch, at Monash, and upon return to RMIT.

The receiver stage architecture is shown in Supplementary Fig. 1. Before photo-detection, the signal was filtered by a programmable optical filter (Finisar WaveShaper 4000 S) set to a 35 GHz passband, in order to select the channel to be measured. The 35 GHz passband was found to be an optimal setting in experiment (see Supplementary Note 1 for more detail). The output of the filter was amplified to ~10 dBm before being directed into a dual-polarisation coherent receiver (Finisar CPDV1200, 43 GHz bandwidth). A local oscillator was provided by an Agilent N7714A laser tuned close to the comb line of interest, at 16 dBm of output power. The photo-detected signals were digitised by the 80-giga-samples-per second (GSa/s), 33-GHz bandwidth inputs of a Keysight oscilloscope (DSO-Z504A, 50 GHz, 160 GSa/s). The digitised waveforms were forwarded to a PC for offline DSP. The DSP flow started with renormalisation, followed by overlap-add chromatic dispersion compensation, then a spectral peak search for frequency offset compensation, followed by frame synchronisation using a short BPSK header, before final equalisation. As the specific fibre types used along the link are not well known, the level of chromatic dispersion compensation was estimated through analysing the header correlation peak height. Equalisation occurred in two stages, with a training-aided least-means-squared equaliser performing pre-convergence, the taps of which were sent to a blind multi-modulus equaliser. After equalisation, a maximum-likelihood phase estimator was used to mitigate phase noise, before the signal was analysed in terms of BER, error-vector magnitude (EVM) and GMI. Further details are included in Supplementary Note 1.

### System performance metrics

After signal reconstruction using DSP, we measured the system performance based on three separate metrics: BER, EVM and GMI.

BER is measured by decoding a 1.1-Mbit-long random bit sequence that was grey-coded onto the 64-QAM constellation. As such, a BER of 9 × 10^−5^ provides 100 errors.

Error-vector magnitude provides an alternative metric, which is directly related to the effective signal-to-noise ratio (SNR) measured at the receiver in the presence of uniform Gaussian noise. We use EVM to calculate signal quality factor (*Q*^2^ [dB]) in Fig. 3 as 20log_10_(1/EVM^2^), with EVM = 1/SNR^0.5^.

In systems employing higher-order modulation formats, GMI provides a more accurate measure of system performance than taking BER and assuming a certain SD-FEC threshold^38^. We use GMI to provide the key performance figures in this demonstration (i.e., net data rate and spectral efficiency). In this case, the achievable capacity (b/s) is calculated as the sum of individual channel GMIs (b/symbol) and multiplying by the symbol rate (symbols/s).

Spectral efficiency (b/s/Hz) can also be calculated from GMI, by taking the mean of the channel GMIs (b/symbol), multiplying by the symbol rate (symbols/second) and dividing by the channel spacing (Hz).

## Supplementary information

Supplementary Information

## Data Availability

The data that support the findings of this study are available from the corresponding authors upon reasonable request.

## Code availability

The algorithms used for this study are standard, and are outlined in detail in the Supplementary Note 1. MATLAB scripts can be provided by the corresponding authors upon reasonable request.
