# Supplementary material for: Ultra-dense optical data transmission over standard fibre with a single chip source
Source: Nat Commun. 2020 May 22;11:2568. doi: 10.1038/s41467-020-16265-x (PMC7244755; doi:10.1038/s41467-020-16265-x)
Supplement: Supplementary file 1 — Supplementary Information [file 41467_2020_16265_MOESM1_ESM.pdf]

Ultra-dense optical data transmission over standard fibre with a single chip source

Bill Corcoran[1], Mengxi Tan,[2] Xingyuan Xu,[2] Andreas Boes,[3] Jiayang Wu,[2] Thach G. Nguyen,[3] Sai T. Chu,[4] Brent E. Little,[5] Roberto Morandotti,[6,7] Arnan Mitchell,[3] and David J. Moss[2]

[1] *Photonic Communications Lab, Department of Electrical and Computer System Engineering, Monash University, Clayton, VIC 3168 Australia*
[2] *Centre for Micro-Photonics, Swinburne University of Technology, Hawthorn, VIC 3122, Australia*
[3] *Integrated Photonics and Applications Centre (InPAC), School of Engineering, RMIT University, Melbourne, VIC 3001, Australia*
[4] *Department of Physics and Material Science, City University of Hong Kong, Tat Chee Avenue, Hong Kong, China.*
[5] *Xi'an Institute of Optics and Precision Mechanics Precision Mechanics of CAS, Xi'an, China.*
[6] *INRS-Énergie, Matériaux et Télécommunications, 1650 Boulevard Lionel-Boulet, Varennes, Québec, J3X 1S2, Canada.*
[7] *Institute of Fundamental and Frontier Sciences, University of Electronic Science and Technology of China, Chengdu 610054, China.*

Supplementary Information

Supplementary Note 1: Experimental set-up

In order to complement the description given in the Methods, a detailed diagram of the experimental set-up is shown in Supplementary Figure 1. The transmission section also shows the control channel used to monitor and control the EDFA hosted remotely in the Monash labs. This is provided by an Ethernet over fibre link, using a FS.com UM-GADF40 media converter at either end of the link. Control and test channel separation is provided by 1310/1550 nm WDM.

We conducted two transmission experiments, sending data over 75 km single mode fibre in the laboratory as well as in a field trial across an installed metropolitan-area single-mode fibre network (see Methods) connecting the RMIT City and Monash Clayton campuses in the greater Melbourne area (Supplementary Figure 2). The transmission link was comprised of two fibre cables connecting labs at RMIT University (Swanston St., Melbourne CBD) and Monash University (Wellington Rd, Clayton). These cables were routed from the labs access panels, to an interconnection point with AARNet's fibre network. The fibre links are a mix of OS1 and OS2 standard cables and include both subterranean and aerial paths. There is no active equipment on these lines, providing a direct dark fibre connection between the two labs. The total loss for these cables was 13.5 dB for the RMIT-Monash link and 14.8 dB for the Monash-RMIT paths. The cable lengths as measured by OTDR were both 38.3 km (totalling 76.6 km in loop-back configuration). At Monash, an EDFA was remotely monitored and controlled using a 1310 nm fibre-ethernet connection running alongside the C-band test channels. The comb was amplified to 19 dBm before launch, at Monash, and upon return to RMIT.

The installed network fiber for the field trial presents a different testing platform to the spooled fibres used in lab [1, 2]. Splices and connections along the link between the two labs provide a source of uncontrolled back-reflections and limit the amount of power that can safely be sent over the network given the risk of connector burns and even fibre fuses from reflective interfaces. Coupled with the higher losses of installed (legacy) fiber links, this provides a challenging platform for high spectral efficiency optical communications where maximising signal to noise ratio is key to enabling high capacities. Moreover, the operation over legacy fibre links covering typical suburban distances demonstrates that it is possible to leverage installed fiber infrastructure for next generation metropolitan/regional area systems, which have been experiencing a higher growth in required capacity than long-haul networks, actually surpassing them in 2017 [3]. This is particularly important due to the cost of laying new fiber in installed ducting being on the order of $30k / mile [1]. It also demonstrates the feasibility of system upgrades using micro-comb-based transceivers to extend the useful lifetime of installed fiber systems.

The comb line OSNR is an important factor in determining the performance of optical frequency combs. We measured the in-band OSNR, accurately using a 150 MHz resolution optical spectrum analyser (Finisar WaveAnalyzer), at several points. We calibrated the WaveAnalyzer against a standard OSA working with 0.1

nm resolution through noise loading a single laser line. Directly after the microcomb, the OSNR was > 33 dB; After the amplifier between the WaveShaper shaping stages (Pritel PM-20-IO), the OSNR was 30 dB, and directly before the modulators on the odd and even 'test-band' arms, it was 28 dB. Note that both after the comb amplifier and directly before the modulators, the optical noise was co-polarized with the comb line. The WaveShapers had an insertion loss of 5dB each in addition to any variable attenuation.

Offline signal generation and reception is performed in MATLAB. The transmitter side defined a frame 2^{18} samples long, to match the memory depth of the Keysight M8195A AWG. The frame consisted of a short sequence of zeros, used for system diagnostics (visually) from the oscilloscope waveforms, followed by a 400 symbol BPSK sequence which was used to denote the start of the data packet. Next, the rest of the samples occupied by a waveform were generated from random integers ranging from 0 to M-1 (where M is the QAM level modulated). The data packet consisted of 64-QAM symbols, where the random data was mapped to the constellation points using Gray coding. The frame was generated at 1 Sa/symbol, then up-sampled with zero padding between symbols to 2 Sa/symbol. The up-sampled waveform was filtered by a root-raised cosine (RRC) filter, set for a roll-off of 2.5% (beta = 0.025), with out-of-band attenuation set to 25 dB (filter defined by 'fdesign.pulseshaping'). This filtered signal was then up-shifted in frequency by 12 GHz. Finally, the shifted signal was resampled to the AWG sample rate. This was done using the 'resample' function that implemented an upsample-FIR-downsample script. The real and imaginary parts of the resulting waveform were sent to the AWG.

When modulating the odd-frequency bands (shifted by +12 GHz from the optical carrier) were generated by adjusting the bias on the CMZM to provide a +90-degree phase shift between the two nested MZMs in that device. For the even channels (shifted by -12 GHz from the optical carrier), this bias was set to -90 degrees. This enabled the normal and inverted outputs of the AWG to be used to generate the two sidebands, which were subsequently delay decorrelated through a length of fibre in the even arm. Independent waveforms were generated for the loading channels, which were modulated with a dual-sideband signal such that the generated loading channels emulated the combination of the odd and even test channels. Given that the electro-optic bandwidth of the Covega Mach-40 modulator was limited (much lower than for SOCNB modulators), we used a pre-emphasis filter to modify the driving waveforms so that a flat spectrum was generated for the loading channels. The pre-emphasis filter was generated as an amplitude-only filter with the filter shape derived from measuring the generated signal spectrum with a 150-MHz resolution optical spectrum analyser (Finisar WaveAnalyzer), and then inverting the measured spectral shape as a pre-emphasis filter. No pre-emphasis was used for the test channels.

Before photo-detection, the signal was filtered by a programmable optical filter (Finisar WaveShaper 4000S) set to a 35 GHz passband, in order to select the channel to be measured. The 35 GHz passband was found to be an optimal setting in experiment. Note that it was not possible for this device to provide a filtering response that matched the channel shape, as these devices typically have a 10 GHz optical transfer function, limiting filter roll-off [4]. Moreover, since we used a 2.5% RRC shaping filter in the transmitter side DSP, if the set receiver side optical filter did not exhibit a flat passband over the signal bandwidth, the signal would have been degraded. Additionally, using too broad a filter would waste receiver dynamic range by partly detecting neighbouring sub-bands [5]. As such, we swept the receiver side filter passband setting, and found that 35 GHz was an optimum value.

The receiver side DSP removed the mean component of the waveforms, running via a Gram-Schmidt orthogonalization method to compensate for receiver I/Q imbalances, followed by overlap/add dispersion compensation with blocks of 1024 samples, and resampled down to 2 Sa/symbol using MATLABs 'resample' function. A spectral peak search was performed to determine the frequency offset by finding the minimal residual carrier left after modulation. The waveform was then filtered by concatenating a static RRC filter to the pulseshaping filter, matching the one used in the transmitter. Frame synchronization was performed by finding the correlation peak between the received waveform and the sent synchronization header. Equalization was then performed using an 81-tap filter. For convenience, we used a least-mean-squares (LMS) algorithm to

initialize a multi-modulus algorithm, as the training-based LMS stage performed source separation without the potential for single polarization convergence. Such a convergence can artificially improve the performance of systems like ours that use delay decorrelation-based polarization multiplexing emulation. We used 10,000 data symbols for training, representing approximately 9% of the total data packet. Note that we were limited in packet size to 4.1 µs (94208 symbols) by the memory depth of our AWG. We expect the channel to remain stable over a longer period of time, such that the training overhead used here would in practice become negligible (for example, a 41 us frame would reduce training overhead to <1%). Moreover, we also post-processed our data using a blind CMA algorithm as a pre-equalization stage, and this resulted in negligible overall performance reduction (less than about 2.5%). Some captures did not converge as well, which we attribute to our particular equalizer implementation and not a fundamental shortcoming of the blind algorithms. We believe that careful optimization of the blind equalization stage would result in negligible penalty compared to the data-aided equalizer. The multi-modulus algorithm worked on a blind radial decision basis, and the filter arose from this algorithm used to equalize the received waveforms. As the DSO and AWG clocks were independent and did not share a common reference, the equalizer was also used as a stand-in for clock recovery. After equalization, a maximum-likelihood based phase estimator was used to provide a phase noise correction, running over a 16-bit averaging window. After phase correction, BER, Q^2 and GMI were calculated. Q^2 was inferred from error-vector magnitude (EVM), as $20.\log_{10}(\mathrm{sqrt}(1/\mathrm{EVM}))$. EVM was calculated from the difference between the magnitude of the sent and received signals. GMI was calculated using the calcGMI.m script provided at https://www.fehenberger.de/#sourcecode.

We also provide additional data on the sensitivity of the 23 Gbd, 64 QAM signal to local oscillator OSNR to illustrate the potential of soliton crystal states to perform as a multiwavelength local oscillator. Supplementary Figure 3 shows the experimental set up and results for the signal quality factor and generalized mutual information (GMI) versus local oscillator OSNR. To make a more direct comparison with our comb, the local oscillator was noise loaded. The OSNR was then measured and filtered by a wavelength selective switch with a bandpass setting of 10 GHz, amplified, and then passed through a polarizing beam splitter before being launched onto the coherent receiver as a local oscillator. We find that there is minimal penalty for the received signal at a comb line OSNR >27 dB, observed in the experiment. We measure a 1 dB penalty in Q2, or a reduction in GMI of 0.3 bits/symbol, which corresponds to an effective rate reduction of about 2.5%.

Supplementary Note 2: Soliton Crystal Generation

While self-localised DKS waves require complex dynamic pumping schemes to initiate, soliton crystals are generated from a fundamentally different process, although both are described by the Lugiato-Lefever equation [6]. Soliton crystals are naturally formed in micro-cavities that display the appropriate form of mode crossings, without the need for the complex dynamic tuning mechanisms that DKS require. They were termed 'soliton crystals', due to their crystal-like profile in the angular domain within the micro-ring resonators [7].

The formation of micro-combs is intimately related to the detuning between the pump wavelength and a resonance of the micro-photonic resonator (e.g. [6, 8]). To generate coherent and low noise micro-combs, the pump wavelength is swept from the blue to the red side of the resonance. This first excites primary combs, typically with line spacings of many resonator FSRs, followed by unstable chaotic combs with high intensity noise, before finally inducing solitons in the resonator. However, these soliton states typically have much lower intra-cavity power than the preceding chaotic states, therefore, as the soliton state is initiated, the resonances shift due to thermal effects and the soliton states become lost. Techniques such as fast wavelength sweeping [9, 10] and power kicking can successfully capture soliton states, but this significantly increases the complexity and footprint of the system due to the need for sophisticated external swept-frequency RF sources and modulators. Deterministic soliton generation cannot generally be achieved by pre-determined tuning into a resonance [11] but requires instead auxiliary stabilization systems [12, 13]. Soliton crystals, on the other hand, are tightly packaged systems of self-localized pulses that have more than ten times higher intra-cavity power than the DKS regime — in fact, close to the power of the spatiotemporal chaotic states [7,14]. Therefore, when sweeping the pump wavelength, switching between chaotic and soliton crystal states does not introduce significant changes in intra-cavity power. Hence, soliton crystals do not suffer from thermal detuning due to

the characteristic 'soliton step' observed for single soliton states. This has the important consequence that they can be initiated through adiabatic pump wavelength sweeping - far simpler than what is required to initiate and maintain DKSs. As such, soliton crystals are highly robust and can provide stable micro-combs without the need for complex feedback systems [14, 15]. This intrinsic robustness is central for enabling the use of micro-combs outside of the laboratory. While soliton crystal micro-combs have been successfully exploited for microwave photonics [15], their potential for coherent optical communications has not yet been reported.

The evolution of a soliton crystal micro-comb while tuning the pump laser manually is shown in the main text as Figure 2 a&b. The open loop generation of the soliton crystal comb [7, 14, 15], along with the low (~50 GHz) line spacing and wide bandwidth of the high conversion efficiency first 'lobe' (4 THz) [15] makes these combs attractive for application to compact, high rate transceivers for optical communications. We note that it is also possible to generate either a different soliton crystal state (Supplementary Figure 4a) or a chaotic state (Supplementary Figure 4b), via slow, manual wavelength tuning. For comparison the actual soliton spectrum used in the experiments is shown again in (Supplementary Figure 4c). The automatic wavelength sweeping over a pre-determined wavelength range (1550.300 – 1550.527 nm) to generate the wanted soliton crystal state was achieved by wavelength tuning the laser in 1pm steps, with half a second between each step. This tuning was achieved by simply setting the wavelength to different values on a Yenista Tunics T100HP, remotely controlling the unit via GPIB through Python. The tuning rate was found through trial and error to allow for reproducible generation of the desired soliton crystal state. While the generation of this state seems robust and repeatable, we make no firm claims to deterministic generation of soliton crystal states, as we have not modelled the generation of the desired state with thermal terms introduced into the LLE, which would allow for the simulation of the effects of thermo-optic chaos [6, 14]. We note that there have been successful demonstrations of deterministic micro-comb generation, although these involve auxiliary systems which greatly increase overall system complexity [12, 13].

In order to estimate the internal conversion efficiency from the pump to comb line within the micro-ring resonator, we analyse the relative powers of the pump line and comb lines emitted from the drop port of the device. Measurement of light from the drop port of our 4-port, dual-bus device accurately reflects the light within the resonator.

We define internal conversion efficiency as the power ratio between the pump line at 1551.05 nm and the comb lines, consistent with [6, 10, 16, 17]. Analysing the spectrum gathered on a standard OSA running at 0.06 nm resolution, we measure an internal conversion efficiency of 42.1%. This compares favourably with dark [16] (20%) and bright [10] solitons (< 0.6%), as expected [7].

Clearly, when taking other factors into account, such as coupling loss, proportion of comb lines used, etc., this will naturally drop, as it will for all devices. We do note that our chips are fibre pigtailed with on-chip mode converters, resulting in extremely low fibre-chip coupling loss < 0.5dB. Further, after selecting the 80 comb lines over the C-band our internal efficiency was 38.1%. This highlights that our soliton crystal state provides the majority of its power (> 90%) in a useful bandwidth for standard C-band optical communications equipment.

The high performance of SCs including both robustness and efficiency has been reported [7, 14] and is understood. It stems from the fact that in SCs the resonator is virtually completely filled with solitons making the intracavity energy very close to the chaotic state, whereas the energy of DKS states largely resides in the CW background rather than the single soliton pulse.

Once established, the robustness of soliton crystal states to relatively large laser frequency drifts was verified by observing that the same stable oscillation state was maintained, and with a maximum power variation of only +2.4 dB to -1.7 dB for the 80 lines used for the transmission experiments even while tuning the pump laser by more than 12 pm (1.5 GHz) – much larger than the actual variation of the pump laser wavelength in the transmission experiments.

We measured the power stability of the comb lines (Supplementary Figure 5) over 66 hours, with spectra captured every 15 minutes with only open loop control (i.e., standard thermo-electric controllers) and without manual intervention to stabilise the micro-ring resonator chip. Supplementary Figure 5a shows the measured spectrum for the 80 C-band comb lines along with the standard deviation (SD in dBm - given by the error bars), showing a relative SD (in dB, S.M. Figure 5b) of about -14dB over the 66 hour period. Conceptually, these measurements demonstrate that the fiber-coupled MRR device can operate as a separate, independent, plug-in element that multiplies the number of coherent carriers produced by an independent laser by 80 times.

We emphasize, however, that in fact it is the net superchannel transmission that is the ultimate test of the comb's performance – not the power stability measurements – and so OSNR is far more important. As long as the required OSNR is maintained, high fidelity data transmission will be supported, which was the case in our experiments. Further, due to the self-stabilizing nature of soliton states in micro-resonators [6, 9], there are fundamental reasons to expect even greater open-loop long-term stability than we have shown in our current measurements. Finally, any improvements to the system such as the addition of feedback control loops, the use of a pump laser with greater frequency stability, better design of the micro-ring resonator thermal stabilization, or the avoidance of comb flattening, would enhance our superchannel transmission performance even further.

We note that this experiment provides the first communication system demonstrated using a soliton crystal state, with previous optical systems demonstrations focusing on applications in microwave photonics [15].

In our experiments we used an external laser source and amplifier to pump the micro-ring used to generate the optical frequency comb. We note that recent demonstrations of hybrid integration of pump lasers to generate single DKS microcombs [18,19], as well as advanced techniques such as injection locking [20], can equally be applied to soliton crystals – in fact probably even more easily given their much simpler generation process.

Hybrid integrated pump sources also yield much greater energy efficiency - the states in [18] were produced with 4 dBm (2.5 mW) pump power. Moreover, the external cavity structure shown in [18] is intrinsically compatible with the much less complex and slower tuning required by soliton crystals.

Integrated laser arrays can now produce high quality optical carriers. Although a full analysis comparing the different approaches is beyond the scope of this work, we make a number of comments. To support tightly spaced superchannel multiplexing with 80 separately integrated lasers with the same performance as micro-combs, it would require precise wavelength locking of all 80 lasers to compensate for variations due to fabrication error. Moreover, the footprint of 80 discrete high-quality lasers may be prohibitively large. Micro-combs, on the other hand, have been realized with hybrid integrated pump sources and have achieved ultralow thresholds <10 mW [18]. Microcombs are intrinsically locked to the micro-resonator free-spectral range, without any feedback requirements. They also have a small footprint, being based on a single high quality resonator and single pump laser source.

Since the nonuniform spectrum of soliton crystal combs has been viewed as a weakness, we chose to flatten the optical frequency comb first so that all lines were of equal power, even though this is not necessary and actually introduces unnecessary impairments – both in our experiments and other micro-comb demonstrations (e.g. [10, 16]). All comb lines are usually wavelength demultiplexed into separate waveguides and sent to separate modulators; it is then straightforward to adjust the comb line power by variable attenuators, amplifiers or even by varying the RF drive amplitude to the modulators.

We implemented comb flattening for several reasons: i) to prove our system operation under the most demanding conditions, ii) to pre-empt the criticism that the nonuniform spectrum of SCs is a limitation, and iii) to facilitate easy comparisons with prior art. Since avoiding flattening would reduce impairments and improve our performance (by increasing the OSNR of the higher power comb lines and their ability to carry

higher spectral efficiency modulation formats, and by eliminating the loss of the extra WaveShaper), it does not represent a limitation to SC based transmission.

Supplementary Note 3: Performance comparisons

Here we provide a wider range of performance comparisons for completeness, against record capacity results in a range of fibres. These results are summarized in Supplementary Table 1.

Reference [10] provides three different system set-ups. The first is closest to our own demonstration, and we use this in the main text for a direct comparison. In this case a single micro-comb is used at the transmitter, and a single laser employed as a local oscillator at the receiver. In the second demonstration in [10], two frequency interleaved combs are used in the transmitter, which, although resulting in a higher overall data rate, resulted in a lower per-comb spectral efficiency, and consequently a lower overall per-comb data rate than the single-comb demonstration. In the third demonstration in [10], a microcomb source is used as a local oscillator in the receiver, with a single comb employed at the transmitter side. In this case, overall system performance was improved to achieve a higher spectral efficiency and hence a higher overall rate, although lower than what we have demonstrated here.

In ref. [16], the same modulation cardinality (i.e. 64 QAM) was used; however, the high comb line spacing and relatively low symbol rate translated to a low spectral occupancy, resulting in a low spectral efficiency and aggregate data rate. The dark soliton state used in this demonstration also seems to require feedback to stabilize the state, increasing system complexity.

The comb source of ref [21] relied on using a benchtop coherent pulse source as a seed for nonlinear broadening. To provide a fully integrated system, this source would need to be on chip. In contrast, soliton-based microcomb generation from an integrated hybrid chip has been demonstrated [18,19]. Although spectral utilization in ref [21] was high, the achieved per-mode spectral efficiency is lower than our demonstration, and used 16-QAM modulation.

For completeness, we also compare against record per-mode results for non-chip based sources - both benchtop-scale and rack mounted comb sources, and with traditional multiple discrete laser based WDM systems.

The highest achieved per-mode data rate from a single comb used a non-integrated rack-mounted, or benchtop fibre-based comb [22]– ie., orders of magnitude larger (and more expensive) than chip-based microcombs, and which has not been realized in integrated form. There, the per-mode capacity across the C & L bands was 97.75 Tb/s, by using high cardinality modulation (64-QAM), high spectral utilization (24.5 Gbd on a 25 GHz grid) and efficient forward error correcting codes. This demonstration indicates that the expansion to the L-band with comb spacings similar to our own can enable even higher aggregate rates.

The highest aggregate rate achieved over single mode fibre [23] used multiple discrete light sources. Again, that demonstration leveraged both C & L bands, although the achieved spectral efficiency is not significantly greater than in our demonstration, even when using 256-QAM relatively high code rate overheads were required (40-50%). This indicates that a single micro-comb source with quality similar to our own can provide performance comparable to multiple discrete laser sources.

We note that the results presented in [23] were achieved using highly specialized fibres and with specialized amplification, neither of which are available in typical networks. The high aggregate rate they present was in part due to the full use of C- and L-bands, compared with the single C-band system we demonstrate here.

Line Rate	Net Rate	SE	Transmission	Source
30.1 Tb/s	28.0 Tb/s	2.8 b/s/Hz	75 km SMF in-lab	Ref. [10], 1st
27.5 Tb/s	25.1 Tb/s	2.6 b/s/Hz	75 km SMF in-lab	Ref. [10], 2nd
37.2 Tb/s	34.6 Tb/s	3.5 b/s/Hz*	75 km SMF in-lab	Ref. [210], 3rd
44.2 Tb/s	40.1 Tb/s	10.4 b/s/Hz	B2B (0 km)	This work
44.2 Tb/s	39.2 Tb/s	10.2 b/s/Hz	75 km SMF in-lab	This work
44.2 Tb/s	39.0 Tb/s	10.1 b/s/Hz	76.6 km SMF installed	This work
4.8 Tb/s*	4.4 Tb/s	0.98 b/s/Hz*	80 SMF km in-lab	Ref. [16]
25.6 Tb/s*	22.0 Tb/s*	3.2 b/s/Hz*	9.6 km, 30-core	Ref. [21]
117.3 Tb/s*	97.75 Tb/s	9.8 b/s/Hz*	31 km, 22-core	Ref. [22]**
174 Tb/s*	120 Tb/s	10.8 b/s/Hz*	70 km 'Z'-type SMF	Ref. [23]***

Note – results from the last two rows are not from chip-based sources but from rack mounted or benchtop based sources and are shown here for completeness.

Supplementary Table 1. Key systems performance metrics, per comb source used in the transmitter, and on a per mode basis. '*' indicates that this figure was not directly provided in the reference, and so is inferred from data provided. '' indicates a demonstration using a commercial benchtop comb source, '***' indicates a traditional WDM result using multiple laser sources.**

Our high spectral efficiency is partly enabled by the baud rate of the channels we modulate. The optimum baud rate in systems where OSNR is not the dominant performance limiter is the subject of on-going research. In systems with high OSNR, noise added by the transmitter and receiver limit performance, either from thermal noise from electrical components, quantization noise from the A/D and D/A converters, as well as noise and distortion from modulation and photodetection. This has proved important for superchannel reception [24,25]. Also, we note that ultra-high spectral efficiency (>15 b/s/Hz) modulation has been based on low baud rates (between 6-10 Gbd) [26,27]. It is on this basis that we suggest that combs with a lower FSR may enable a higher spectral efficiency, which should also improve the overall single-device data rate. The trade-off is that a reduction of line spacing results in a lower power per-line, impacting the individual comb line OSNR (if the same total bandwidth is preserved), which may ultimately pose a limit to the spectral efficiency as has been noted [28]. Understanding this trade-off is the subject of on-going research.

Supplementary Figures

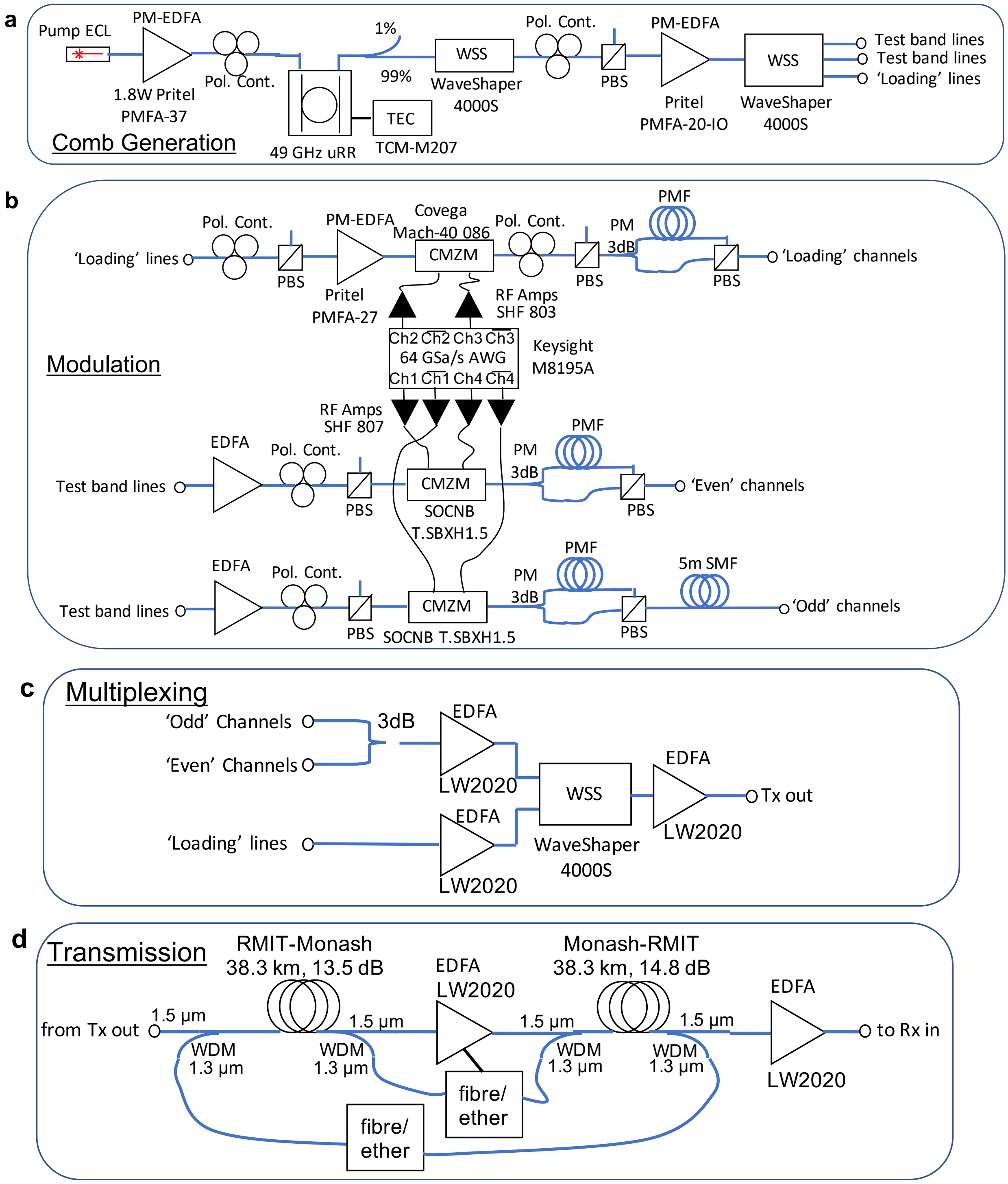

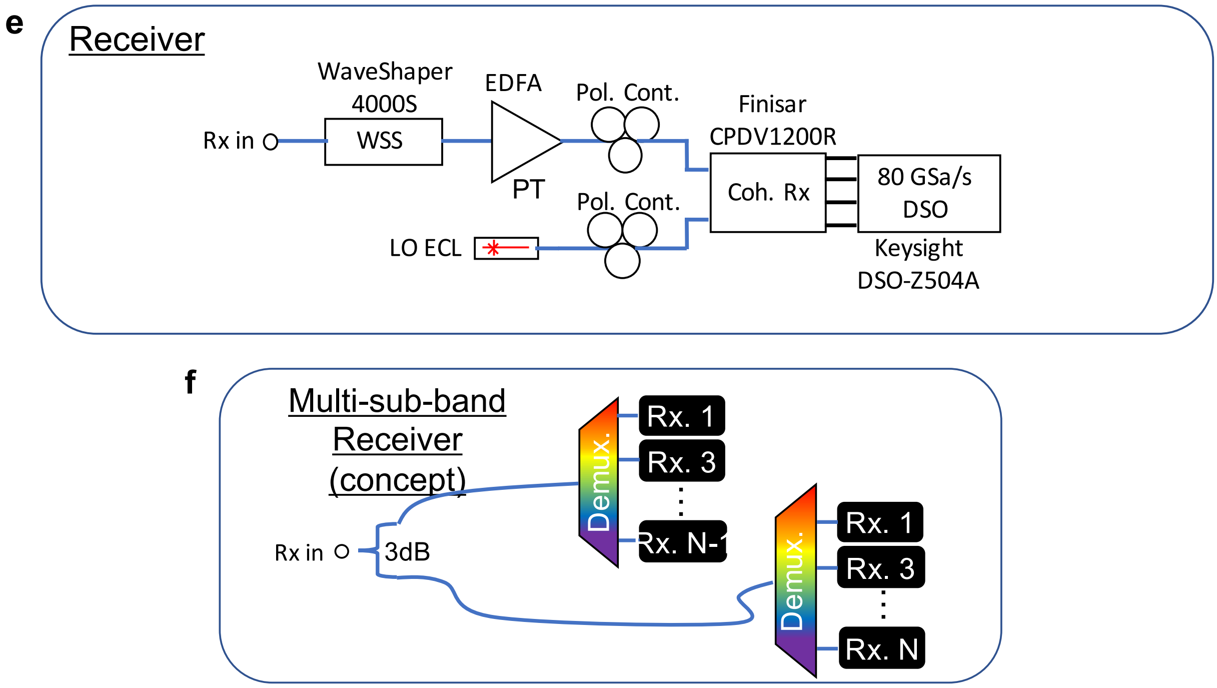

Supplementary Figure 1 - A detailed diagram of the experimental set-up. ECL: External cavity laser, PM-EDFA: Polarization maintaining EDFA, Pol. Cont.: Polarization controller, uRR: micro-ring resonator, TEC: Thermo-electric controller, WSS: Wavelength selective switch, PM 3dB: Polarization maintaining 50%/50% optical power coupler/splitter, PBS: Polarization beam splitter, PMF: Polarization maintaining fibre, CMZM: Complex Mach-Zehnder modulator (dual nested type), AWG: Arbitrary waveform generator, RF Amps.: microwave amplifiers, WDM: Wavelength division multiplexer (1310/1550 nm), fibre/ether: Gigabit Ethernet to fibre channel media converter, Coh. Rx: integrated optical hybrid and balanced photodiode coherent receiver, DSO: Digital storage oscilloscope (real-time). For the EDFAs, LW2020 denotes Lightwaves2020 MOABF17 optical amplifiers, PT denotes a Photonic Technologies 'Powerflat' amplifier. f) depicts the concept of a receiver for every individual sub-band.

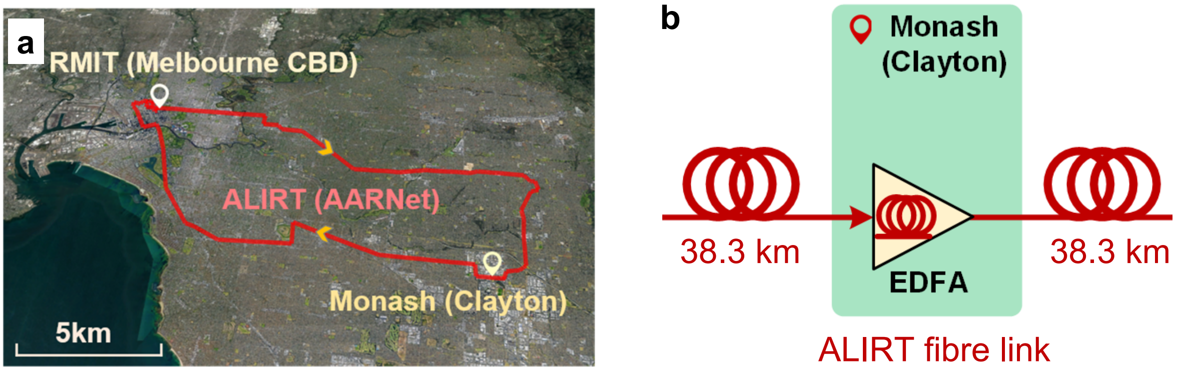

Supplementary Figure 2 – Field trial test link. a) Layout of the field trial link, linking RMIT and Monash labs via AARNet's fibre network, with route overlayed on Google Maps (red). Projection of the path of the installed fibre cabling onto a composite aerial image of the greater Melbourne metropolitan area (image courtesy of Google Maps). The fibre route included a mix of aerial and sub-surface fibres, comprising both OS1 and OS2 standard cables, and was provided by AARNet as part of the ALIRT project. b) Simplified schematic of fibre link, two 38.3 km fibre spans running along the same path with a remote EDFA at the Monash labs.

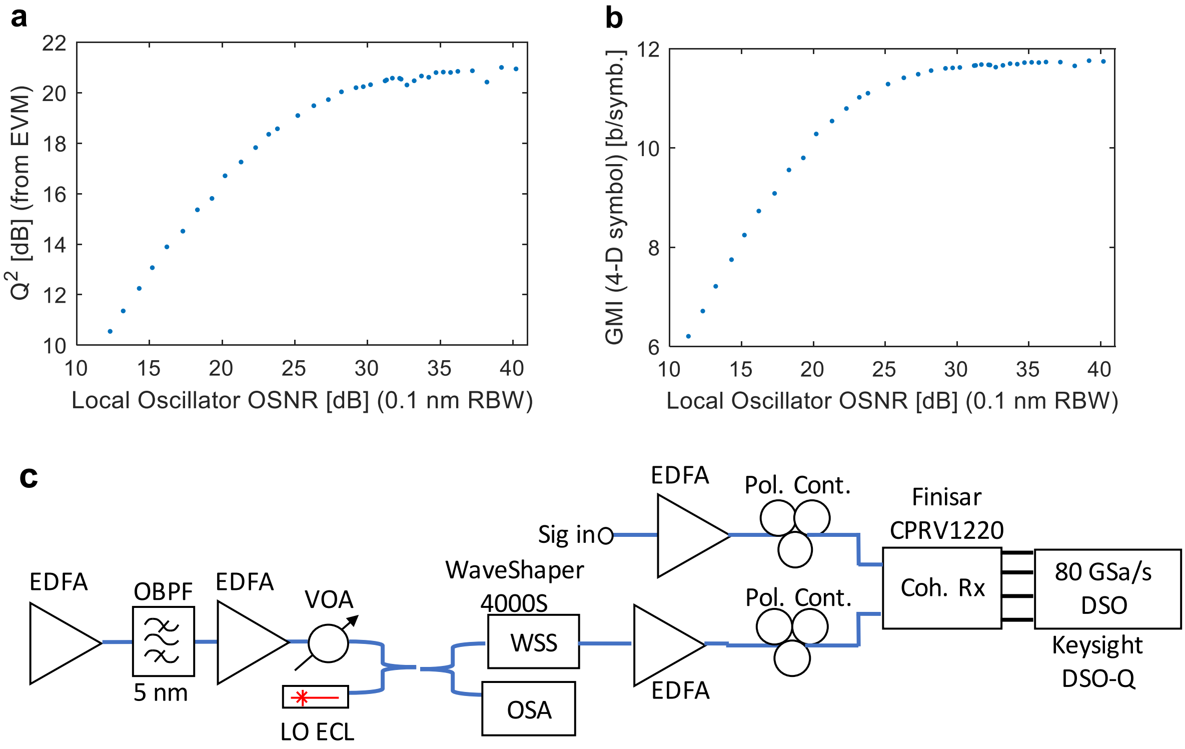

Supplementary Figure 3 – Signal tolerance to local oscillator (LO) optical signal to noise ratio (OSNR). a) Signal quality factor (Q^2, as extracted from error vector magnitude as $20\log_{10}[1/EVM^2]$) and b) generalized mutual information per 4-D symbol (GMI) against local oscillator quality factor. OSNR is loaded onto the local oscillator, then filtered by a WSS set to provide a 10 GHz passband, as shown as c). This filtering set-up mirrors that used for the frequency comb. OBPF: Optical band pass filter, WSS: Wavelength Selective Switch, EDFA: Erbium-doped fibre amplifier, ECL: External cavity laser, OSA: Optical spectrum analyser, DSO: Digital Sampling Oscilloscope, Coh. Rx: Coherent receiver.

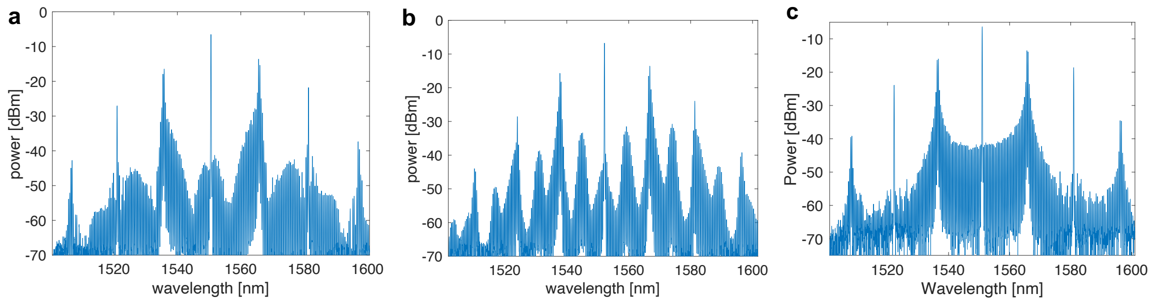

Supplementary Figure 4 – Alternate comb generation states. a) Measured spectrum of an alternate soliton crystal generation state achieved through manual wavelength tuning of the pump laser. b) Captured spectrum of a modulation instability ('chaotic') state, also achieved via manual wavelength tuning. c) Final complete soliton crystal spectrum used in the transmission experiments.

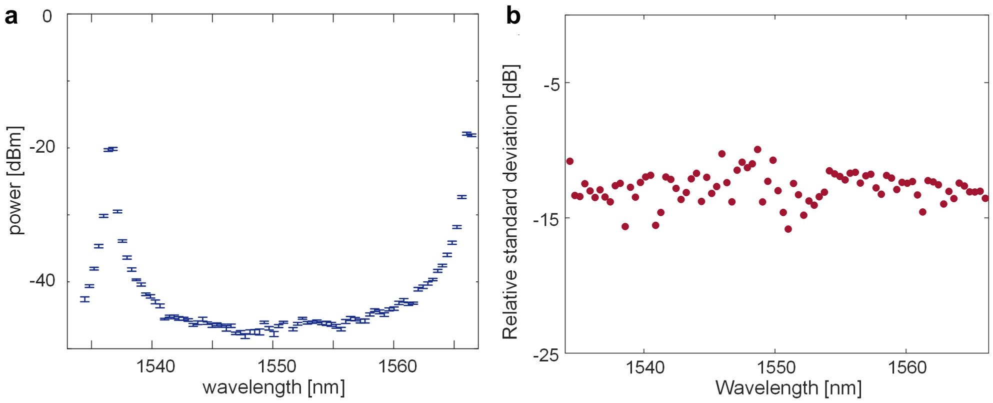

Supplementary Figure 5 – Power stability measurements. a) zoomed in soliton spectrum of the 80 channels selected over the C-band for the experiments, along with the power standard deviation (error bars) of the comb lines over 66 hours (with traces captured at 15 minute intervals), and b) relative power standard deviation (in dB).

Supplementary References

[1] S. Grubb, et al., Field trials: Is it make or break for innovative technologies?, *Proc. OFC 2018*, S2A (2018)

[2] Shuto, Y., et al., Fiber fuse phenomenon in step-index single-mode optical fibers, *J. Quantum Electron.*, **40**, 1113-1121 (2004)

[3] Cisco Corp., Cisco Visual Networking Index: Forecast and Trends, 2017–2022 White Paper, available at https://www.cisco.com/c/en/us/solutions/service-provider/visual-networking-index-vni/index.html#~complete-forecast (accessed 6 Aug. 2019)

[4] C. Pulikkaseril et al., Spectral modelling of channel band shapes in wavelength selective switches, *Opt. Express*, **19**, 8458-8470 (2011)

[5] Y. Mori et al., Wavelength-division demultiplexing enhanced by silicon-photonic tunable filters in Ultra-Wideband Optical-Path Networks, *J. Lightwave Technol.*, DOI 10.1109/JLT.2019.2947709 (2019)

[6] Pasquazi, A., et al., Micro-Combs: A Novel Generation of Optical Sources, Physics Reports 729, 1–81 (2018).

[7] Cole, D. C., Lamb, E. S., Del'Haye, P., Diddams, S. A. & Papp, S. B., Soliton crystals in Kerr resonators. Nat. Photonics 11, 671–676 (2017).

[8] Herr, T. et al., Temporal solitons in optical microresonators. Nat. Photonics 8, 145–152 (2013).

[9] Gaeta, A.L., Lipson, M., Kippenberg, T.J., Photonic-chip-based frequency combs, Nature Photonics 13, 158-169 (2019).

[10] Marin-Palomo, P. et al., Microresonator-based solitons for massively parallel coherent optical communications, Nature 546, 274–279 (2017).

[11] Bao, C., et al., Direct soliton generation in microresonators, Opt. Lett, 42, 2519-2522 (2017).

[12] Zhou, H., et al., Soliton bursts and deterministic dissipative Kerr soliton generation in auxiliary-assisted microcavities, *Light: Science and Applications*, **8**, 50 (2019)

[13] Kang, Z., et al., Deterministic generation of single soliton Kerr frequency comb in microresonators by a single shot pulsed trigger, *Opt. Express,* **26**, 18563-18577 (2018)

[14] Wang, W., et al.., Robust soliton crystals in a thermally controlled microresonator, Opt. Lett., 43, 2002-2005 (2018).

[15] Xu, X., et al., Photonic microwave true time delays for phased array antennas using a 49 GHz FSR integrated micro-comb source, *Photonics Research*, **6**, B30-B36 (2018)

[16] Fülöp, A., et al., V., High-order coherent communications using modelocked dark-pulse Kerr combs from microresonators, Nat. Commun., 9, 1598 (2018).

[17] Bao, H., et al., Laser Cavity Soliton Micro-Combs, Nat. Photon., 13, 384–389 (2019).

[18] Stern, B, et al., Battery-operated integrated frequency comb generator, *Nature,* **562,** 401-406 (2018)

[19] Raja, A.S., et al., Electrically pumped photonic integrated soliton microcomb, *Nat. Comms.,* **10**, 680 (2019)

[20] Pavlov, N.G., Narrow-linewidth lasing and soliton Kerr microcombs with ordinary laser diodes, *Nat. Photon.*, **12**, 694–698 (2018)

[21] Hu, H. et al., Single-source chip-based frequency comb enabling extreme parallel data transmission, Nat. Photon., 12, 469–473 (2018).

[22] Puttnam, B., et al., 2.15 Pb/s Transmission Using a 22 Core Homogeneous Single-Mode Multi-Core Fiber and Wideband Optical Comb, Proc. European Conference on Optical Communications (ECOC), PDP 3.1, Valencia (2015)

[23] Ionescu, M, et al., 91 nm C+L hybrid distributed Raman–erbium-doped fibre amplifier for high capacity subsea transmission, *Proc. ECOC 2018,* doi:10.1109/ECOC.2018.8535151 (2018)

[24] R. Maher, Signal processing for high symbol rate transmission: Challenges and Opportunities, *Proc. Adv. Photonics* 2018, SpW3G.2 (2018)

[25] L. Galdino et al, The trade-off between transceiver capacity and symbol rate, *Proc. OFC 2018*, W1B.4 (2018)

[26] Olsson, S.L.I, et al., Record-high 17.3-bit/s/Hz spectral efficiency transmission over 50 km using probabilistically shaped PDM 4096-QAM, *Proc. OFC 2018*, Th4C.5 (2018)

[27] Chen, X., et al., 16384-QAM transmission at 10 Gbd over 25-km SMF using polarization-multiplexed probabilistic constellation shaping, *Proc ECOC 2019*, PD.3.3 (2019)

[28] Torres-Company, V., et al.., Laser Frequency Combs for Coherent Optical Communications, J. Lightwave Technol. doi: 10.1109/JLT.2019.2894170 (2019).
